# Radiofrequency catheter ablation of ventricular arrhythmias arising from the region above pulmonary valve

**DOI:** 10.1186/s12872-019-1220-2

**Published:** 2019-10-22

**Authors:** Jin Li, Cheng Zheng, Zhi-Rui Liu, Jun Ma, Ge Jin, Wei-Qian Lin, Yao-Yao Wang, Jia-Feng Lin

**Affiliations:** 1 Department of Cardiology, the Second Affiliated Hospital and Yuying Children’s Hospital of Wenzhou Medical University, 109 Xueyuan Road, Wenzhou, 325000 Zhejiang China; 2 Department of Cardiology, Ruian People’s Hospital and the Third Affiliated Hospital of Wenzhou Medical University, Wenzhou, China

**Keywords:** Ventricular arrhythmias, PSCs and MSPA, Radiofrequency catheter ablation, ECG characteristics, Electrophysiological characteristics

## Abstract

**Background:**

Ventricular arrhythmias (VAs) arising from the origin above pulmonary valve lack comprehensive investigation. This study aimed to disclose the characteristics and radiofrequency catheter ablation (RFCA) outcomes for those VAs.

**Methods:**

One hundred six VAs arising from the region above pulmonary valve treated with RFCA were included in this study.

**Results:**

Seventy-five cases were identified in the pulmonary sinus cusps (PSCs, 32 in left sinus cusp (PLC), 15 in right (PRC), 28 in anterior (PAC)) and 31 cases were in the main stem of pulmonary artery (MSPA, 18 above PLC (LMSPA), 3 above PRC (RMSPA), 10 above PAC (AMSPA)). Compared with PSCs VAs, MSPA VAs exhibited a higher R wave amplitude in the inferior leads, a total inferior R amplitude > 5.1 mV predicting MSPA origins. LMSPA, RMSPA and AMSPA VAs resembled PLC, PRC and PAC VAs in electrocardiographic characteristics respectively. No electrophysiological differences were found between PSCs and MSPA VAs. The irrigated-up catheter and R0 Swartz long sheath were more utilized for ablation of PSCs VAs than for MSPA VAs. All these VAs were successfully eliminated by RFCA.

**Conclusion:**

VAs arising from the origin above pulmonary valve were common. Based on certain electrocardiographic characteristics, they could be roughly located, which contributed to an effective RFCA.

## Background

Most premature ventricular contractions/ ventricular tachycardia (PVCs/VT) exhibiting left bundle branch block (LBBB) morphology with inferior axis were identified arising from the right ventricular outflow tract (RVOT) and could be effectively eliminated by radiofrequency catheter ablation (RFCA) [1–5]. Whereas, a minority of PVCs/VT with these electrocardiographic feathers were found arising from the anatomic structures in the vicinity of RVOT, such as left ventricular outflow tract (LVOT), main stem of pulmonary artery (MSPA) and pulmonary sinus cusps (PSCs) [6–11]. Incomprehensive understandings of the relationship between the electrocardiographic characteristics of ventricular arrhythmias (VAs) and the complicated anatomic structures always led to an unsuccessful ablation. In this study, we retrospectively reviewed and analyzed 106 PVCs/VT exhibiting left bundle branch block (LBBB) morphology with inferior axis, which were finally eliminated by RFCA in the region above the pulmonary valve (including MSPA and PSCs). The specific electrocardiographic and electrophysiological characteristics of these PVCs/VT were explored and the effect of RFCA for these PVCs/VT were also evaluated.

## Methods

### Study population

From January 2010 to May 2017, 1416 patients with frequent PVCs/VT received RFCA treatment in the cardiac electrophysiological center of the Second affiliated hospital and Yuying Children’s Hospital of Wenzhou medical university. Among these patients, 106 PVCs/VT (7.5%) were confirmed arising from the region above the pulmonary valve by selective pulmonary artery angiography combined with 3-dimension mapping system. All these 106 PVCs/VT were successfully eliminated by RFCA and included in this study.

### Electrocardiographic analysis

Before RFCA, in each patient, 24-h holter was carried out at least once. The ECG was monitored for another 24 h by ECG telemetry just prior to catheter ablation. All these ECG monitoring consistently showed the PVC count ≥10,000/24 h. The 12-lead electrocardiogram (ECG) of each PVCs/VT were obtained before RFCA, the QRS morphology of PVCs/VT were analyzed. No patients showed T wave inversion in precordial leads or epsilon wave behind the QRS in lead V1. The ECG index of PVCs/VT below were analyzed: 1) QRS amplitude in each lead; 2) QRS morphology in each lead (q, r, s refer to amplitude less than 0.5 mV, Q,R,S refer to amplitude more than 0.5 mV); 3) Precordial transition zone; 4) Precordial transition index (the precordial transitional zone of PVCs/VT minus it of sinus rhythm).

### Electrophysiological study and RFCA

All anti-arrhythmias drugs were discontinued at least for 5 half-lives before electrophysiological study and RFCA. All patients underwent the standard electrophysiological study and RFCA. Catheters were delivered into the cardiac ventricle under fluoroscopy via the femoral vessels. If clinical arrhythmias didn’t occur spontaneously, isoproterenol infusion (2 to 5 mg/min) in addition to programmed electrical stimulation was performed to provoke clinical arrhythmias. The protocol consisted of ventricular stimulation at 3 basic drive cycle lengths (600, 500, 430 ms respectively) with < 2 extra stimuli to a minimum coupling interval of 230 ms. In patients with frequent PVCs or VT, 3-dimensional electroanatomical mapping was used. Point-by-point mapping was performed using a steerable 7.5-F, D-curve catheter with a 3.5-mm tip electrode to create anatomic maps. Activation mapping combined with pace mapping was used to identify the origin during PVCs or VT in the study. The target site for ablation was defined as sites mapped with the earliest ventricular activation and exhibiting good pace match (≥10/12 lead match). Once a target site was located, RFCA application was attempted. When a target site was mapped near the pulmonary artery, pulmonary arteriography was performed before ablation to investigate the distance from the tip of ablation catheter to those structures. Pulmonary arteriography was performed using a right Judkins 4.0 catheter located above pulmonary valve, the PLC was seen situated relatively left and at the lowest level among three PSCs, LMSPA referred to the area above PLC; the PAC was situated in the superior and anterior position, AMSPA referred to the area above PAC; and the PRC was situated in the superior and relatively right position, RMSPA referred to the area above PRC. The catheter tip ablating on target site was monitored in right anterior oblique (RAO) and left anterior oblique (LAO) projection during both systole and diastole by pulmonary arteriography to confirm a tight contact.

When the temperature-controlled catheters were utilized, radiofrequency applications were delivered with a target temperature of 50–55 °C and maximum power output of 50 W, the impedance during energy application was expected between 80~140 Ω. If the impedance produced by temperature-controlled catheters was too high, the irrigated-tip catheters with a 17 ml/min saline flow rate were utilized instead, with the goal to achieve a target temperature of 43 °C and maximum power output of 30–35 W. If the target site was found in the pulmonary sinus cusps, reversed U curve of the ablation catheter can facilitate the stability of a mapping catheter within the PSCs. The long sheath was always applied to stabilize the mapping catheter and help it rotation in the 3 individual PSCs. If the VT or PVCs accelerated or decelerated during the first 10s of energy application on the target sites, the radiofrequency delivery was lasted for 60 to 180 s. If not, the radiofrequency delivery was ceased, and another target site was searched for. Pulmonary arteriography was needed to be repeated after the successful ablation to reconfirm the specific location of target sites. Programmed electrical stimulation and intravenous isoproterenol were both performed before withdrawal all catheters and sheaths to reassure the complete elimination of PVCs/VT.

### Definition of success

Acute success was defined as disappearance of spontaneous or provoked clinical VAs at the end of the procedure and during the first 24 h post-operation off any antiarrhythmic drugs. Long-term success was defined as absence of PVCs/VT or at least an 75% decrease in PVC burden during 24-h Holter 3 months latter without taking any anti-arrhythmic drugs.

### Follow-up

Each patient was followed up in our hospital’s outpatient department at 1 month, and every 3 months thereafter by cardiologists. Transthoracic echocardiography and 24-h Holter monitoring were performed on the day after the procedure and during each follow-up. Whenever the symptom of VAs recurred, the 12-lead ECG and 24 holter were examined immediately.

### Ethnics of the study

This retrospective study was approved by the institutional review board of the Second Affiliated Hospital and Yuying Children’s Hospital of Wenzhou Medical University. Each patient provided the written informed consent for the procedure of electrophysiological study and RFCA.

## Results

### Basic clinical characteristics of studied population

The 106 patients (32males), average aged 45.99 ± 13.66 years old, had a mean history course of 3.15 ± 1.07 years. Twenty-four hours Holter before RFCA showed the average PVCs counts of 21,318.00 ± 7262.01 beats/24 h. No patients were found with structural heart diseases by physical examination and a series of laboratory examinations. According to the location of target sites relative to the pulmonary artery, the VAs further divided into two subgroups, the MSPA (main stem of pulmonary artery) group and the PSCs (pulmonary sinus cusps) group. The MSPA group consisted of 31 patients (10 males and 21 females), average age of 44.51 ± 13.61 years old. In MSPA group, 18 cases (58.06%) showed target sites located above pulmonary left sinus cusp (PLC) with an average distance of 11.44 ± 5.91(8~35) mm to the bottom of PLC, 10 cases (32.26%) located above pulmonary anterior sinus cusp (PAC) with a distance of 12.34 ± 6.65(5~53) mm above the bottom of cusp, 3 cases (9.68%) located above pulmonary right sinus cusp (PRC) with a distance of 10.28 ± 3.61 (8~16) mm to the bottom of cusp. The PSCs group consisted of 75 patients (21 males and 54 females), average age of 46.60 ± 13.29 years old. Among 75 patients, 32 cases (42.67%) were ablated at the PLC, 28 cases (37.33%) at the PAC, 15 cases (20%) at PRC. Detail basic clinical characteristics of these patients were concluded in the Table 1.

**Table 1:** Basic clinical data of patients

Basic Clinic Data	Total (*n* = 106)	MSPA (*n* = 31)	PSCs (*n* = 75)	*P* value
Age (Y)	45.99 ± 13.66	44.51 ± 13.61	46.60 ± 13.29	> 0.05
Sex (Male)	32	10 (32.25)	22 (30.14)	> 0.05
Smoking	11	3 (9.68)	8 (10.95)	> 0.05
Hypertension	11	3 (9.68)	8 (10.95)	> 0.05
Diabetes	9	3 (9.68)	6 (8.22)	> 0.05
Arrhythmias (PVCs)	89	26 (83.87)	63 (84.00)	> 0.05
LVEDD (mm)	44.20 ± 7.05	46.08 ± 7.81	43.43 ± 6.61	> 0.05
LVEF (%)	62.37 ± 6.57	61.45 ± 7.15	62.76 ± 6.33	> 0.05
Counts/24 h	21,318.00 ± 7262.01	24,602.52 ± 7833.80	19,960.46 ± 6600.23	> 0.05
Disease course (Y)	3.15 ± 1.07	3.04 ± 1.07	3.19 ± 1.08	> 0.05

### Electrophysiological study and radiofrequency ablation

**Fig. 1:**
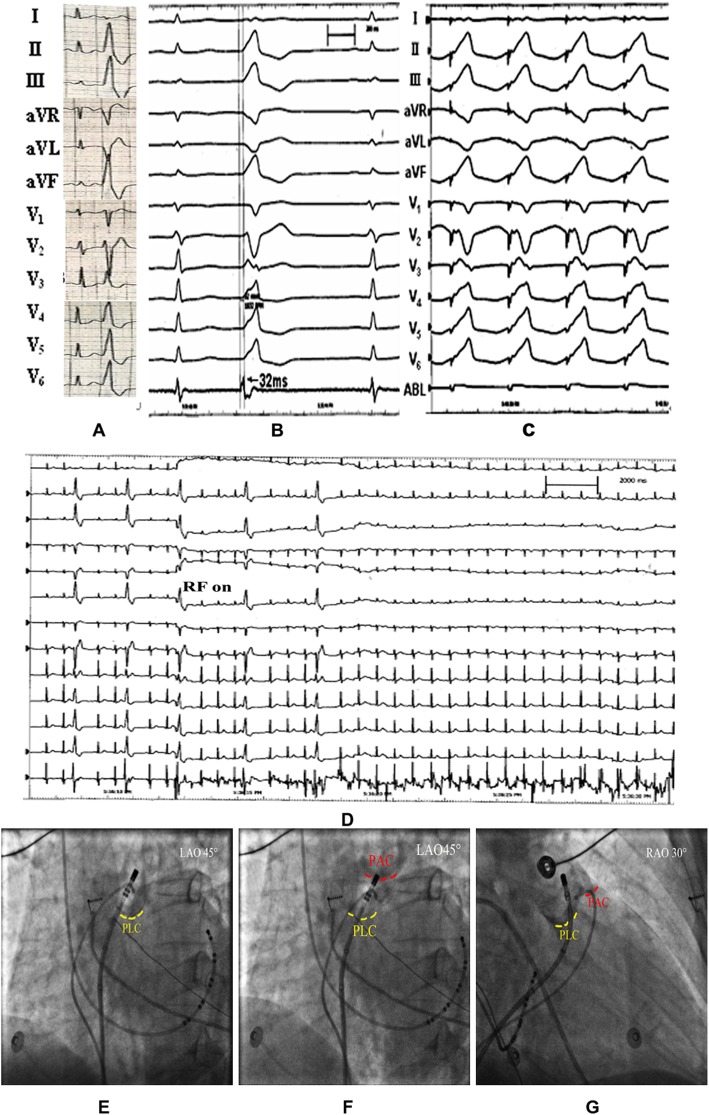
Radiofrequency catheter ablation of PVCs rising from MSPA 16 mm above PLC. **a** ECG of the PVCs. The PVCs exhibited a left bundle branch block (LBBB) morphology and inferior axis deviation, r morphology in lead I, QS morphology in both aVL and aVR with QS_aVR_>QS _aVL_, rS pattern in lead V1-V2, R morphology in lead II, III, aVF and V4-V6 with an ascending notch, precordial transition zone between lead V2-V3. High R wave was recorded in inferior leads with an average amplitude more than 3.0 mV. **b** Activation mapping of the PVCs. Activation mapping in MSPA above PLC showed a local ventricular activation with initial discrete potential preceding the QRS onset by 32 ms. **c** Pace mapping of the target site. Pacing mapping performed on the site with earliest ventricular activation showed an excellent pace match between paced QRS and clinical PVCs. **d** PVCs disappeared after energy application on target site for 6 s. **e, f** Left anterior oblique projection of the target site (the tip of the catheter). **g** Right anterior oblique projection of the target site (the tip of the catheter)

**Fig. 2:**
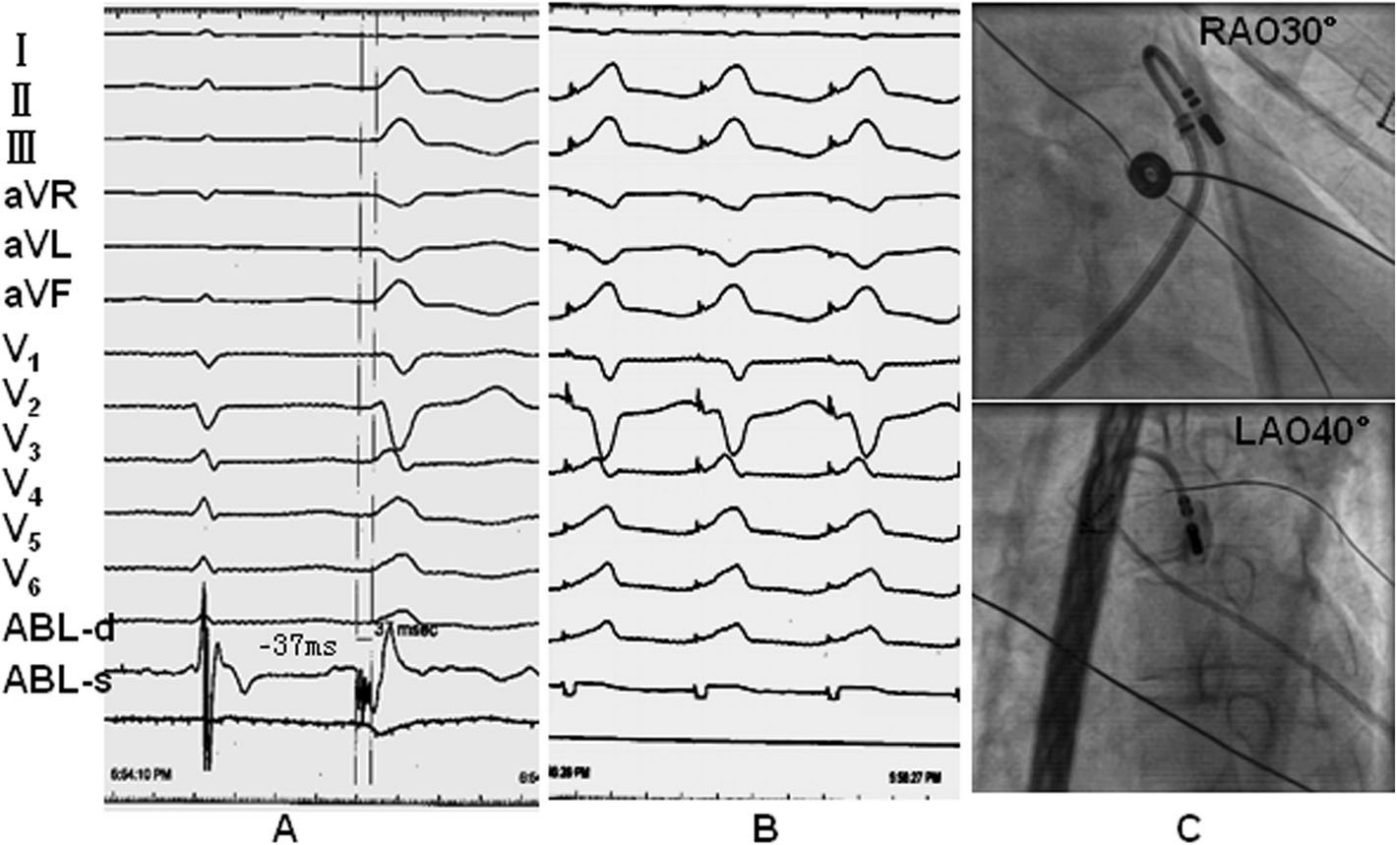
Radiofrequency catheter ablation of PVCs rising from PAC. **a** Activation mapping of PVCs in the PAC. The PVCs exhibited a left bundle branch block (LBBB) morphology and inferior axis deviation, rs morphology in lead I, QS morphology in both aVL and aVR with QS_aVL_>QS _aVR_, rS pattern in lead V1-V2, R morphology in lead II, III and aVF and V4-V6, precordial transition zone between lead V2-V3. Activation mapping showed local ventricular activation with initial discrete peak potential preceding the QRS onset by 37 ms. **b** Pace mapping showed a 11-lead match between paced QRS and clinical PVCs. **c** Angiograph of pulmonary artery prior to ablation showed the tip of ablation catheter located in anterior PSCs. Energy application in PAC led to instant elimination of PVCs. The PVCs could not be induced by further isoproterenol administration combined with programmed electrical stimulation. No recurrence was reported during the follow-up of 2 years

Among these 106 PVCs/VT, the target site for ablation was determined based on pace mapping combined with activation mapping. Pace mapping showed good pace match in 95 cases (89.62%), in which complete match of 12 leads was obtained in 40 cases (42.11%), 11-lead match obtained in 33 cases (34.73%), 10-lead match in 22 cases (23.16%). Activation mapping showed that in bipolar electrogram, the earliest ventricular activation of target sites preceding the QRS onset by average of 35.05 ± 6.24 ms. Among 106 cases, far-field atrial potential was recorded in 63 PVCs/VT (59.43%), pulmonary artery potential was recorded in 18 PVCs/VT (16.98%), merged or discrete local ventricular potential of peak or split morphology was record in 69 PVCs/VT (65.09%) with 53 cases (50.00%) exhibiting a reversion of potential (the local ventricular potential preceding the ventricular electrogram during VAs was recorded in the middle of or after the ventricular electrograms during sinus rhythm). In unipolar electrogram, all target sites exhibited QS morphology with 41 cases pesented a downward notch in desecnding limb of QS. There was a slight difference of pace-match leads between MSPA VAs and PSCs VAs (MSPA 11.34 ± 0.52 leads match versus PSCs 11.08 ± 0.90 leads match, *P* < 0.05), no other significant differences were found based on electrophysiological investigation. The procedure of catheter ablation was further compared between MSPA group and PSCs group. In MSPA group, 26 cases (83.87%) were ablated by temperature-controlled catheter while 5 cases (16.13%) by irrigated-tip catheter. On the contrary, most cases (66, 88.00%) in PSCs group were ablated by irrigated-tip catheter, while a few cases (9, 12.00%) were ablated by temperature-controlled catheter. Ablation catheter supported with R0 Swartz long sheath was needed in majority cases of PSCs origin during the procedure (71, 94.67%), while only needed in 2 cases of MSPA origin (2, 6.45%). In addition, there were more procedural time, fluoroscopic time and energy delivery time required for VAs arising from MSPA than VAs from PSCs (*p* < 0.05). Besides, more lesions of application were required for MSPA VAs than PSCs VAs (*P* < 0.05). Comparison between MSPA VAs and PSCs VAs on electrophysiological study and RFCA was shown in Table 2. Typical cases of VAs arising from MSPA and PSCs were showed in Fig. 1-2**.**

**Table 2:** Electrophysiology study and Radiofrequency catheter ablation of VAs arising from MSPA and PSCs

	Total (*n* = 106)	MSPA (*n* = 31)	PSCs (*n* = 75)	*P* valve
Pulmonary artery potential	18 (17.0%)	4 (12.9%)	14 (18.7%)	> 0.05
QS morphology in unipolar electrogram	106 (100%)	31 (100%)	75 (100%)	> 0.05
With notch in descending limb	41 (38.7%)	11 (35.5%)	30 (40%)	> 0.05
Farfield atrial potential of target site in bipolar electrogram	63 (59.4%)	18 (58.1%)	45 (60.0%)	> 0.05
Merged or discrete potential of peak or split morphology of target site in bipolar electrogram	69 (65.1%)	20 (64.5%)	49 (65.3%)	> 0.05
Reversed polarity of bipolar electrograms	53 (50.0%)	15 (48.4%)	38 (50.7%)	> 0.05
Application of temperature-controlled catheter	34 (32.1%)	26 (83.87)	9 (13.0)	< 0.001
Application of irrigated-tip catheter	71 (67.0%)	5 (16.13)	66 (87.00)	< 0.05
V-QRS (ms)	−34.71 ± 4.26	−35.62 ± 6.79	−34.78 ± 3.75	> 0.05
Pace match of target site (lead)	11.16 ± 0.90	11.34 ± 0.52	11.08 ± 0.90	< 0.05
Pacing target site induced good pace match	95 (89.6%)	25 (80.7%)	70 (93.3%)	> 0.05
12-lead match	40 (37.7%)	10 (32.3%)	30 (40.0%)	> 0.05
11-lead match	33 (31.1%)	9 (29.0%)	24 (32.0%)	> 0.05
10-lead match	22 (20.8%)	6 (19.4%)	16 (21.3%)	> 0.05
Application of R0 Swartz sheath	73 (68.9%)	2 (6.45%)	71 (94.67)	< 0.001
Procedural time (min)	54.28 ± 13.63	65.24 ± 17.26	46.28 ± 9.30	< 0.001
Fluoroscopic time (min)	8.07 ± 2.93	9.31 ± 3.16	7.19 ± 2.77	< 0.05
Lesions of energy application	2.76 ± 1.89	3.89 ± 2.59	2.20 ± 0.79	< 0.001
Energy delivery duration (s)	243.86 ± 88.40	335.32 ± 72.11	210.76 ± 65.80	< 0.001
Success of ablation	106 (100%)	31 (100.00)	75 (100.00)	> 0.05
Response of energy application on target site				
PVC/VT disappeared immediately	95 (89.6%)	27 (87.10)	68 (90.67)	> 0.05
PVC accelerated or decelerated prior to disappearance	11 (10.4%)	4 (12.90)	7 (10.72)	> 0.05

### ECG characteristics of the VAs arising from the region above the pulmonary artery

In all patients, 12-lead ECG during VAs exhibited a typical LBBB morphology with an inferior axis. There were 4 VAs showing precordial transition zone in lead V1, 7 VAs in lead V2, 11 VAs between lead V2-V3; 51 VAs in lead V3; 25 VAs between lead V3 and V4, 7 VAs in lead V4, 1 VAs in lead V4-V5. A total of 12 cases presented a precordial transitional zone index < 0. The ECG characteritics of VAs arisng from MSPA and PSCs were concluded in Table 3. Compared with the PSCs VAs, MSPA VAs showed relatively higher R wave amplitude in the inferior leads (*p* < 0.05). R wave amplitude in the inferior leads> 5.1 mV indicated an MSPA origin, with 96.55% sensitity and 72% specificity (area under the curve [AUC] = 0.907). There were no significant differences on R/S Ratio in lead V1 and V2, precordial transition zone, precordial transition index between MSPA and PSCs VAs (*p* > 0.05).

**Table 3:** ECG characteristics comparison between VAs arising from MPSA and from PSCs

Origin	MPSA (*n* = 31)	PSCs (*n* = 75)	*P*
R_II_ (mV)	2.32 ± 0.52	1.81 ± 0.58	< 0.05
R_III_ (mV)	2.36 ± 0.65	1.69 ± 0.55	< 0.05
R_aVF_ (mV)	2.34 ± 0.58	1.74 ± 0.56	< 0.05
R_II_+ R_III_+ R_aVF_ (mV)	6.76 ± 1.38	4.62 ± 1.04	< 0.05
V1 R/S	0.21 ± 0.07	0.28 ± 0.05	> 0.05
V2 R/S	0.31 ± 0.12	0.35 ± 0.07	> 0.05
Precordial transition zone			
< V3	7 (22.58%)	15 (20.00%)	> 0.05
= V3	15 (48.39%)	36 (48.00%)	> 0.05
> V3	9 (29.03%)	24 (32.00%)	> 0.05
Precordial transition index			
> 0	12 (38.71%)	26 (34.67%)	> 0.05
= 0	16 (51.61%)	40 (53.33%)	> 0.05
< 0	3 (9.67%)	9 (12.00%)	> 0.05

The 106 VAs were further divided into 3 groups based on their target sites relative to each PSC and its corresponding MSPA, VAs arising from the pulmonary anterior sinus cusp (PAC) and the MSPA above PAC group (PAC-AMSPA, 38 cases), VAs arising from the pulmonary left sinus cusp (PLC) and the MSPA above PLC group (PLC-LMSPA, 50 cases) and VAs arising from the pulmonary right sinus cusp (PRC) and the MSPA above PRC group (PRC-RMSPA, 18 cases). The ECG characteristics of VAs arising from each PSCs and its corresponding MSPA were shown in Table 4. VAs from PAC-AMSPA (38 cases) usually exihibited QS, qs, rs, qr. or r pattern in lead I, predominant R wave in inferior leads, deeper negative wave in aVL than aVR, dominant R wave in V4-V6. VAs from PLC-LMSPA (50 cases) usually exihibited rs or r pattern in lead I, predominant R wave in inferior leads, deeper negative wave in aVR than aVL, dominant R wave in V5-V6. VAs from PRC-RMSPA (18 cases) usually exihibited a R pattern in lead I, predominant R wave in inferior leads, deeper negative wave in aVR than aVL, dominant R wave in V5-V6.

**Table 4:** The ECG characteristics of VAs arising from each PSC and its corresponding MSPA

	Anterior PSC and corresponding MSPA (PAC-AMSPA, 38cases)	Left PSC and corresponding MSPA (PLC-LMSPA, 50 cases)	Right PSC and corresponding MSPA (PRC-RMSPA, 18 cases)
Lead I	QS、qs、rs、qr. or r pattern	rs or r pattern	R pattern
Lead II, III and aVF	R pattern, ascending limb notch or peak notch may be seen	R pattern, ascending limb notch or peak notch may be seen	R pattern, descending limb notch may be seen
Lead aVR and a VL	QS, QS_aVL_>QS_aVR_	QS, QS_aVR_>QS_aVL_	main QS, a minority of qs or qR, QS_aVR_>QS_aVl_
Lead V1-V2	rS or RS	rS or RS	rS or RS
Lead V3	rS, RS or Rs	rS, RS or Rs	rS
Lead V4	R or Rs	R or Rs	Rs or R
Lead V5-V6	R pattern, ascending limb notch or peak notch may be seen	R pattern, ascending limb notch or peak notch may be seen	R pattern, descending limb notch may be seen
R _I_ (mV)	−0.12 ± 0.11^#^^	0.36 ± 0.13	0.41 ± 0.18
R_II_ (mV)	1.92 ± 0.48	1.83 ± 0.38	1.45 ± 0.41^*#^
R_III_ (mV)	2.04 ± 0.49^#^^	1.52 ± 0.42*^	1.11 ± 0.39^*#^
R_aVF_ (mV)	1.96 ± 0.47^#^^	1.67 ± 0.39*^	1.27 ± 0.41^*#^
R_II + III + aVF_ (mV)	6.02 ± 1.45^#^^	5.13 ± 1.32*^	3.94 ± 1.01^*#^
QS_aVR_ (mV)	−0.96 ± 0.22	−1.05 ± 0.36	− 0.92 ± 0.39
QS_aVL_ (mV)	−1.07 ± 0.26^#^^	−0.61 ± 0.32*^	− 0.33 ± 0.28^*#^
QS_aVR/aVL_	0.89 ± 0.23^#^^	1.72 ± 1.32*^	2.79 ± 2.57^*#^
RV1(mV)	0.28 ± 0.13	0.32 ± 0.12	0.36 ± 0.22
RV2(mV)	0.35 ± 0.17	0.55 ± 0.22*^	0.42 ± 0.24
RV3(mV)	0.63 ± 0.31	0.89 ± 0.37*^	0.66 ± 0.33
RV4(mV)	1.28 ± 0.52	1.39 ± 0.51	0.99 ± 0.41^*#^
RV5(mV)	1.79 ± 0.53	1.68 ± 0.51	1.38 ± 0.54^*#^
RV6(mV)	1.72 ± 0.43	1.67 ± 0.53	1.36 ± 0.49^*#^
RV4 + V5 + V6(mV)	4.79 ± 1.32	4.74 ± 1.68	3.73 ± 0.82^*#^

*Compared with PAC-AMSPA, *p* < 0.05

^#^Compared with PLC-LMSPA, *p* < 0.05

^Compared with PRC-RMSPA, *p* < 0.05

### Follow up

Acute success of RFCA was achieved in all 106 patients. No procedure-related complications occurred. The patients were followed up for 24.2 ± 18.6 (3–60) months without taking antiarrhytmic drugs. The clinical PVCs/VT were absent on 24 h-Holter 3 months post ablation in 98 patients. Four patients had at least a 75% decrease in the PVC burden, among whom, the initial ablation produced a small change in the QRS morphology in 2 patients. Long term failure of ablation was reported on 4 patients, but the morphology of PVCs/VT was completely different from the previous ones.

## Discussion

Recently, idiopathic ventricular arrhythmias have been demonstrated arising from specific anatomic structures above pulmonary valve, including PSCs and MSPA, which resembled the idiopathic VAs arising from RVOT in QRS morphology [9–11]. For VAs of left bundle branch block morphology and inferior axis, when failed to be eliminated by RFCA in ventricular outflow tract, further mapping and ablation should be attempted in area above pulmonary valve, including PSCs and MSPA. In this study, we found when dividing the VAs arising from the area above the pulmonary valve into PSCs and MPSA groups, it was challenging to differentiate VAs of PSCs from those of MPSA due to similar ECG characteristics and electrophysiological characteristics. Whereas, when we divided the VAs arising from the area above pulmonary valve into PAC-AMSPA, PLC-LMSPA and PRC-RMSPA according to their origin relative to each PSC and the MSPA above it, some ECG differences could be identified to distinguish these three entities, which may help locate the ectopy of VAs and facilitate the RFCA procedure.

**Fig. 3:**
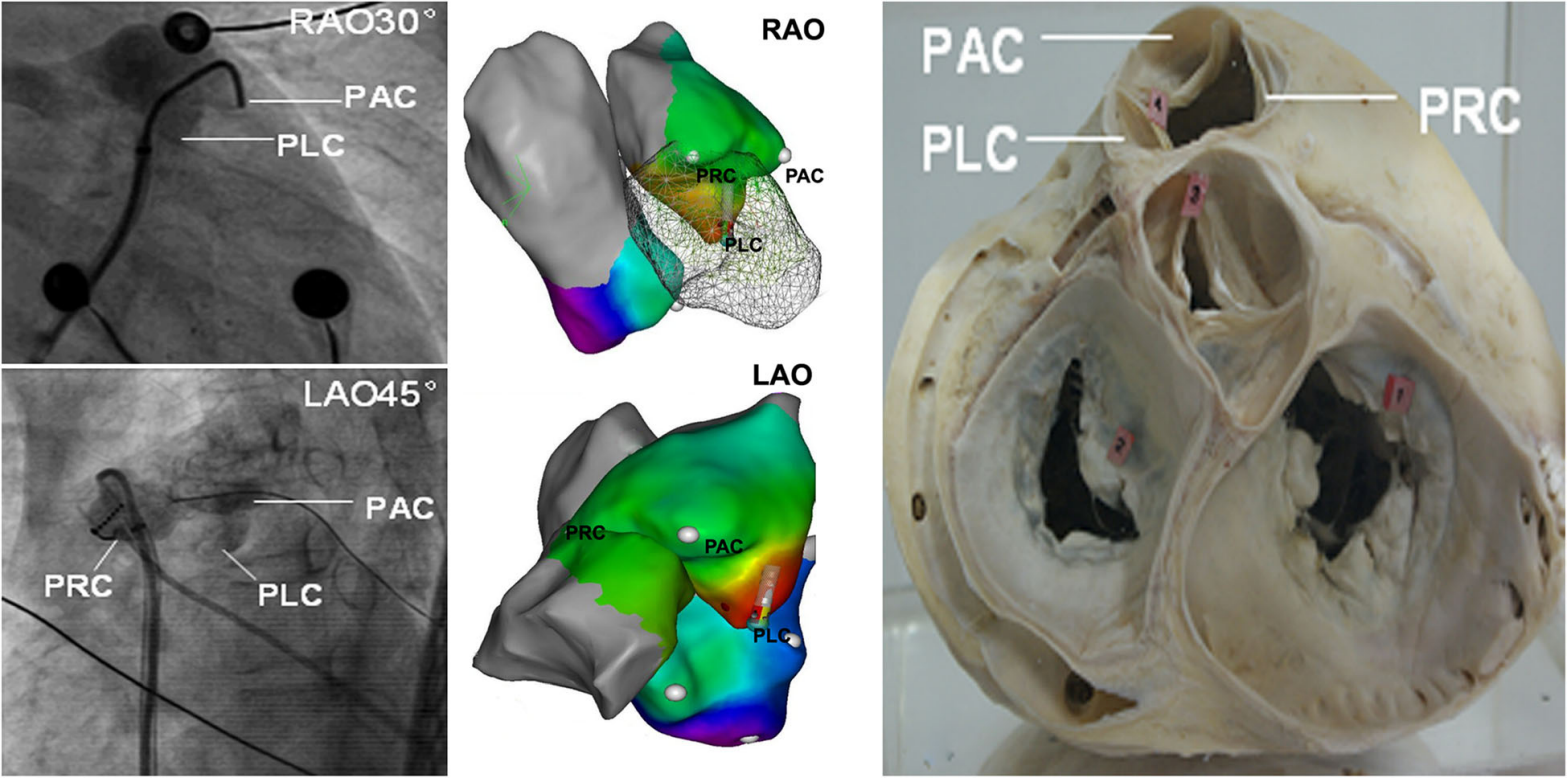
PLC-LMSPA, PAC-AMSPA and PRC-RMSPA anatomy. Pulmonary arteriography, three-dimension mapping and RVOT anatomy revealed that the PLC-LMSPA is situated at the lowest level above the posterior septum of the RVOT, the PAC-AMSPA situated relatively superior above the anterior septum of RVOT and PRC-RMSPA situated at the most rightward above the free wall of RVOT

The pulmonary valve not only divided the right ventricular outflow tract from the pulmonary artery, but also lead to the formation of three pulmonary sinus cusps. Current anatomical study revealed that RV musculature could extend to the pulmonary root above pulmonary valve in a sleeve-like manner, more distally into each PSC and the MSPA above it. It has been demonstrated that the myocardial extension with wide proximal portion attaching the RVOT, tampering at the distal end in PSCs or MSPA, could be the substrate of ventricular arrhythmias [12–14]. Because of the existence of myocardial extension from RVOT to each PSC or to its corresponding MSPA, it was reasonable to speculate that the VAs arising from each PSC resembled the VAs from the MSPA above it. By the interdigitation of the 3 PSCs, the pulmonary root above the pulmonary valve could be divided into PAC-AMSPA, PRC-RMSPA and PLC-LMSPA, see Fig. 3.

### Prevalence of VAs arising from the area above pulmonary area

Several studies have reported the prevalence of VAs arising from the area above pulmonary. Tada et al. found 4% PVCs/VT arising from the pulmonary artery (PA), and the distance from the origin to the base of the pulmonary valve, was less than 15 mm in 64% of patients [11]. ZiLi Liao et al. reported that VAs with a PSC location were noted in 24 (11%) of 218 idiopathic outflow tract–like VAs treated by using RFCA [9]. In the present study, 7.5% (106/1416) PVCs/VT were found arising from the region above the pulmonary valve. Taken these data together, we thought VAs arising from the area above the pulmonary valve was not a seldom phenomenon, and suggested that the area above the pulmonary valve should be routinely mapped when failed ablation in RVOT occurred.

### Mapping and RFCA in the area above pulmonary valve

The target site of VAs in the current study were identified by the earliest ventricular activation, which preceded the QRS onset by 34.71 ± 4.26 ms (MSPA35.62 ± 6.79 versus PSCs 34.78 ± 3.75, *p* > 0.05), and pace mapping, which resulted at least 10-lead match in 89.6% patients (MSPA 80.7% versus PSCs 93.3%, *p* > 0.05). Several studies have investigated the specific potentials that could be used to identify the target sites of VAs arising from the area above pulmonary valve. Tada et al. reported a sharp potential recorded within the PA during the PVCs/VT and (or) sinus rhythm was a characteristic of PA-PVCs/VT and was considered to be the myocardial extension in PA. The existence sharp potential could be used to guide ablation of PA-VAs [11]. Zili L et al. reported that in VAs arising from PSCs, 2 local ventricular potentials were record at target site, the first potential during VAs was recorded after the ventricular electrograms during sinus rhythm [9]. In our study, the target sites were commonly recorded with far-field atrial potential, pulmonary artery potential and potential reversion. However, none of these potentials can be used to distinguish PSCs from MSPA origin.

During the RFCA procedure of VAs arisng from the area above the pulmonary artery, the application of irrigated-tip catheter and R0 Swartz long sheath was needed in majority cases of PSCs group, which was in consistency with previous studies. Due to the relative slow blood flow in the PSCs, utilization of irrigated-tip catheter would ensure sufficient energy delivery and effective ablation. With the support of SR0 long sheath, the ablation catheter can be easily reversed U curved and stably rotated to access 3 individual PSCs, a better contact between the catheter tip and target site could be achieved.

### ECG findings

The ECG characteristic of VAs arising from the area above the pulmonary valves have been explored by several studies. A previous study suggested that PA-VAs can be distinguished with RVOT with larger R-wave amplitude in the inferior ECG leads, aVL/aVR ratio of the Q-wave amplitude, and R/S ratio in lead V2 [12]. Zili Liao et al. has once investigated the electrocardiographic characterisitcs of VAs originating from the pulmonary sinus cusps, and concluded that VAs of PRC had significantly larger R-wave amplitude in lead I and a smaller aVL/aVR ratio of Q-wave amplitude, meanwhile, the R-wave amplitude in inferior leads was smaller in VAs localized in the PRC than the PAC and PLC [9]. In this study, based on a much larger sample size investigated, we further concluded the specific ECG characteristics of VAs arising from the area above the pulmonary valve. First, we found that when comparing the whole MSPA VAs with the whole PSCs VAs, except a larger R-wave amplitude in inferior leads, no other significant differences were noted. Second, we found VAs arising from each PSCs showed similar ECG characteristics with those arising from the MSPA above it. Thus, we further divided the VAs above pulmonary valve into PAC-AMSPA, PLC-LMSPA, PRC-RMSPA groups. We concluded that VAs arising from PAC-AMSPA exhibiting a relative lower R wave amplitude in lead I, higher R wave amplitude in inferior leads, smaller aVR/aVL when compared with PRC-RMSPA and PLC-LMSPA. PRC-RMSPA were distinguished from PLC-LMSPA and PAC-AMSPA by smaller negative wave in lead aVL, larger aVR/aVL, lower R wave amplitude in inferior and lateral leads. The VAs arising from PLC-LMSPA usually exhibited a larger R wave amplitude in V2 and V3.

The ECG characteristics of VAs arising from each PSC and the MSPA above it can be rationally explained by their specific anatomical structure. As the PAC-AMSPA is located above the anterior septum of RVOT, which was more superior, anterior area of the pulmonary artery root, VAs arising from this ares usually depolarized downward and posteriorly along the ventricular septum in the relatively opposite direction from lead I, resulting in a more negative QRS morphology in lead I and a much higher R wave amplitude in inferior leads. PRC-RMSPA is located above the freewall of RVOT, VA arising from this area alway depolarized downward to excited the RVOT free wall and then progressively to involve the ventricle septum and left ventricle, which contributed to a much lower R wave amplitude in inferior leads and lateral leads. The PLC-LMSPA was located above posterior septum approximately approaching the left aortic sinus cusp, VAs arising from this ares usually depolarized downward and anteriorly along the septum toward the V2 and V3 leads, thus a much higher R wave amplitude in V2-V3 can be found.

### Conclusion

Based on our current research, we concluded when VAs of LBBB morphology and inferior axis failed to ablation in ventricular outflow tract, further mapping and ablation should be attempted in the area above pulmonary valve. The ectopy of VAs above the pulmonary valve could be roughly located based on the ECG characteristics, which may facilitate the RFCA procedure and improve successful ratio of RFCA treatment.

## Data Availability

The datasets analyzed during the current study available from the corresponding author on reasonable request.

## Abbreviations

AMSPA: Main stem of pulmonary artery above PAC
ECG: Electrocardiogram
LBBB: Left bundle branch block
LMSPA: Main stem of pulmonary artery above PLC
LVOT: Left ventricular outflow tract
MSPA: Main stem of pulmonary artery
PAC: Pulmonary anterior cusp
PLC: Pulmonary left cusp
PRC: Pulmonary right cusp
PSCs: Pulmonary sinus cusps
PVCs: Premature ventricular contractions
RFCA: Radiofrequency catheter ablation
RMSPA: Main stem of pulmonary artery above PRC
RVOT: Right ventricular outflow tract
VAs: Ventricular arrhythmias
VT: Ventricular tachycardia
